# All Nations Depend on the Global Knowledge Pool – Analysis of Country of Origin of Studies Used for Health Technology Assessments in Germany

**DOI:** 10.1371/journal.pone.0059213

**Published:** 2013-03-14

**Authors:** Kirsten H. Herrmann, Robert Wolff, Fueloep Scheibler, Siw Waffenschmidt, Lars G. Hemkens, Stefan Sauerland, Gerd Antes

**Affiliations:** 1 Institute for Quality and Efficiency in Health Care, Cologne, Germany; 2 German Cochrane Centre, University Hospital Freiburg, Institute of Medical Biometry and Medical Informatics, Department of Medical Biometry and Statistics, Freiburg, Germany; 3 Kleijnen Systematic Reviews Ltd, York, United Kingdom; 4 Basel Institute for Clinical Epidemiology and Biostatistics, University Hospital Basel, Basel, Switzerland; University of New South Wales, Australia

## Abstract

**Background:**

Health Technology Assessments (HTAs) are used to inform decision-making and their usefulness depends on the quality and relevance of research and specific studies for health-policy decisions. Little is known about the country of origin of studies used for HTAs.

**Objective:**

To investigate which countries have made the largest contributions to inform health policy decisions through studies included in HTAs in Germany.

**Methods:**

The country of origin was extracted from all studies included in HTAs of the German Institute for Quality and Efficiency in Health Care, (IQWiG), published from 2/2006 to 9/2010. Studies were ranked according to the total number of studies per country, adjusted for population size, gross domestic product (GDP), and total health expenditure.

**Results:**

1087 studies were included in 54 HTA reports. Studies were assigned to 45 countries. Most of the studies (27%) originated from the United States (USA), 18% were multinational, followed by 7% from the United Kingdom (UK) and 5% from Germany. Nordic countries led the ranking when adjusting for population size/million (ranks 1-3,6,9/45 countries), GDP/billion US$ (1,2,5,9,14/45), or health expenditure/billion US$ (1,3,5,12,13/45). The relative contribution of the UK was stable in the analyses when adjusted for population size (7/45), GDP (7/45), and health expenditure (9/45), whereas the USA (13, 18, and 30/45) and Germany (17, 19, and 21/45) dropped in the ranking.

**Conclusions:**

More than half of the studies relevant for evidence-informed decision-making in Germany originated from the USA, followed by multinational research and the UK. Only 5% of the studies originated from Germany. According to our findings, there appears to be some discrepancy between the use of globally generated evidence and the contribution to the knowledge pool by individual countries.

## Introduction

Health Technology Assessment (HTA) is a multidisciplinary field that systematically investigates the clinical efficacy and effectiveness, safety, cost, cost-effectiveness of health care interventions, as well as organizational implications, social consequences, and legal and ethical considerations [1]. HTA plays a crucial role in health systems throughout the world, supporting decision-making on how to access, distribute and implement technologies and innovation. Health technologies include pharmaceuticals, devices, diagnostics and treatments, and other clinical, public health, and organizational interventions. HTA reports include a systematic review of the clinical evidence in a transparent, unbiased, and robust manner in order to quantify the potential benefits and risks of health technologies [2], [3].

The Federal Joint Committee (G-BA) and the Institute for Quality and Efficiency in Health Care (IQWiG) are the most relevant HTA institutions in Germany. G-BA is the central self-governing body within the German health care system, a committee comprising important stakeholders in the health care system, e.g. health care providers and statutory health insurance funds. ‘It issues directives for the benefit catalogue of the statutory health insurance funds for more than 70 million insured persons and thus specifies which services in medical care are reimbursed’ [4]. IQWiG is an independent scientific institute and investigates the benefits and harms of medical interventions, usually to inform the decisions made by the G-BA [5].

A variety of publications are available on the analysis of the research activities of different countries. These are mainly bibliometric analyses focusing on Cochrane reviews [6] or specific medical fields such as anesthesia [7], [8], dentistry [9], nuclear medicine [10], pharmacological trials [11], primary care [12], radiology [13] or surgery [14].

In addition to these scientific analyses, inclusion in an HTA report emphasizes and measures the impact of clinical research on evidence-informed decision-making and thus the value in research expenditure as well as the return on investment in patient-oriented research. Consideration of studies for HTA reports indicates the quality and relevance of research for health-policy decisions. However, little is known about the country of origin of studies used for HTAs. It is therefore of interest to analyze which countries conduct studies used for HTAs and hence provide research findings relevant to health-policy decisions.

The objective of our study is to investigate the country of origin of clinical studies included in HTAs in a specific country, using Germany as an example.

## Methods

### Search strategy

The IQWiG website www.iqwig.de, which provides an overview of IQWiG projects from 2004 onwards, was searched in September 2010 for completed HTA reports. IQWiG products comprise benefit assessments (full reports and rapid reports), working papers, appraisals of clinical practice guidelines (CPGs), as well as health information. Products pursuant to § 35a of the German Social Code Book V (assessments of dossiers submitted by pharmaceutical companies) and health economic evaluations were not considered in this analysis, as these types of documents were only published after our project had been completed. Both full reports and rapid reports are HTAs applying the same methods with regard to the actual content of the report. However, they differ in terms of procedures, e.g. in contrast to rapid reports, preliminary versions of full reports are discussed in a public hearing. Working papers provide information on relevant developments in health care or methodological issues. Health information (e.g. feature articles, fact sheets and research summaries) is produced to inform the general public. Appraisals of CPGs aim to describe current health care standards. The latter three types of products are not considered to be HTAs [5].

### Inclusion criteria

Eligible HTA reports were benefit assessments (full reports and rapid reports) published on the IQWIG website between 2004 and September 2010.

To be included in an HTA report prepared by IQWiG, studies need to contain data relevant to the specific project, i.e. report patient-relevant outcomes such as mortality, morbidity, adverse events and quality of life related to the health technology under investigation [5]. In our analysis we considered all studies meeting the inclusion criteria of the HTA reports. No further criteria were applied.

### Exclusion

We excluded IQWiG products not considered to be HTA reports, such as working papers, health information articles or appraisals of CPGs.

### Data extraction

Clinical studies were ascribed to a country as obtained from data extraction tables in published HTA reports. The HTAs reported the country of origin of the studies included based on the location of the study center(s) of the specific study. In cases where this was unclear to the HTA authors, the origin of a study was classified as “unknown”. A study was classified as “multinational” in cases where multiple study centers in various countries (or even a continent) were reported. Neither multinational studies nor those of unclear geographic origin were considered for the adjustments of the subsequent analyses. The overall number of studies included in the HTA reports from one country was used in the analysis and calculated in Excel 2010 (Microsoft Corporation, USA). A ranked order was displayed for further comparisons and analyses.

### Quality assessment

Inclusion in an HTA report was used as a quality indicator. These reports are conducted following a rigorous assessment of the risk of bias (e.g. assessment of study design, allocation concealment) and follow recognized standards of evidence-based medicine. They typically included randomized controlled trials (RCTs) for the evaluation of clinical effects, as well as other study designs (e.g. diagnostic accuracy studies) [5]. We did not conduct any additional quality assessments of the primary studies included in the HTA reports.

### Analysis

We analyzed how the studies included were distributed across countries adjusted for size of population (studies per 1 million population), gross domestic product (GDP, studies per 1 billion US$ GDP) and national spending on health (studies per 1 billion US$ health expenditure).

Data from the International Monetary Fund (2010) [15] were extracted to adjust for GDP. To adjust for the size of population and total health expenditure, data from the World Health Organization (WHO) report ‘World Health Statistics 2010’ [16] were obtained. Data for the Republic of China (ROC) were not included in the WHO report and therefore obtained from the Central Intelligence Agency report ‘The World Factbook’ 2010 [17] and ‘Lists of countries by total health expenditure 2007’ [18].

For a more detailed analysis we divided the reports into those on drugs and those on non-drug interventions. As an example, the rankings of countries of origin were analyzed according to health expenditure.

## Results

81 projects were finalized and published between 06/02/2006 and 11/09/2010. 54 completed HTA reports (full and rapid reports) were included. 26 of these were reports on drugs and 28 on non-drug interventions (table 1), e.g. surgical procedures and diagnostic devices. 27 projects were guideline appraisals, working papers and health information and therefore excluded from the analysis (table 2).

**Table 1:** Appendix 1. Reports included.

	Project No	Title
	Year	Link
1.	A04-01A	Exenatide - Diabetes mellitus Typ 2 - Rapid Repor
	2006	https://www.iqwig.de/language-selector.986.en.html?tid=1117&phlex_override_command=element
2.	A04-01B	Clopidogrel plus acetylsalicylic acid in acute coronary syndrome
	2009	https://www.iqwig.de/a04-01b-clopidogrel-plus-acetylsalicylic-acid-in.986.en.html?tid=1202&phlex_override_command=element
3	A04-02	L-methionine in patients with neurogenic bladder disorders
	2010	https://www.iqwig.de/a04-02-l-methionine-in-patients-with-neurogenic.986.en.html?tid=1201&phlex_override_command=element
4	A05-01	Long-acting insulin analogues in the treatment of diabetes mellitus type 1
	2010	https://www.iqwig.de/a05-01-long-acting-insulin-analogues-in-the.986.en.html?tid=1197&phlex_override_command=element
5	A05-02	Rapid-acting insulin analogues in the treatment of diabetes mellitus type 1
	2007	https://www.iqwig.de/a05-02-rapid-acting-insulin-analogues-in-the.986.en.html?tid=1195&phlex_override_command=element
6	A05-03	Long-acting insulin analogues in the treatment of diabetes mellitus type 2
	2009	https://www.iqwig.de/a05-03-long-acting-insulin-analogues-in-the.986.en.html?tid=1194&phlex_override_command=element
7	A05-04	Rapid-acting insulin analogues in the treatment of diabetes mellitus type 2
	2006	https://www.iqwig.de/a05-04-rapid-acting-insulin-analogues-in-the.986.en.html?tid=1192&phlex_override_command=element
8	A05-05A	Glitazones in the treatment of diabetes mellitus type 2
	2009	https://www.iqwig.de/a05-05a-glitazones-in-the-treatment-of-diabetes.986.en.html?tid=1191&phlex_override_command=element
9	A05-05C	Glinides in the treatment of diabetes mellitus type 2
	2009	https://www.iqwig.de/a05-05c-glinides-in-the-treatment-of-diabetes.986.en.html?tid=1187&phlex_override_command=element
10	A05-08	Urine and blood glucose self-measurement in diabetes mellitus type 2
	2009	https://www.iqwig.de/a05-08-urine-and-blood-glucose-self-measurement.986.en.html?tid=1152&phlex_override_command=element
11	A05-09	Different antihypertensive drugs as first-line therapy in patients with essential hypertension
	2009	https://www.iqwig.de/a05-09-different-antihypertensive-drugs-as-first.986.en.html?tid=1151&phlex_override_command=element
12	A05-13	Fixed combinations of corticosteroids and long-acting beta-2-receptor agonists for inhaled use in patients with asthma
	2007	https://www.iqwig.de/a05-13-fixed-combinations-of-corticosteroids-and.986.en.html?tid=1147&phlex_override_command=element
13	A05-14	Leukotriene receptor antagonists in patients with asthma
	2006	https://www.iqwig.de/a05-14-leukotriene-receptor-antagonists-in.986.en.html?tid=1146&phlex_override_command=element
14	A05-19A	Cholinesterase inhibitors in Alzheimer's disease
	2007	https://www.iqwig.de/a05-19a-cholinesterase-inhibitors-in-alzheimer-s.986.en.html?tid=1141&phlex_override_command=element
15	A05-19B	Ginkgo compounds in Alzheimer's disease
	2008	https://www.iqwig.de/a05-19b-ginkgo-compounds-in-alzheimer-s-disease.986.en.html?tid=1139&phlex_override_command=element
16	A05-19C	Memantine in Alzheimer's disease
	2009	https://www.iqwig.de/a05-19c-memantine-in-alzheimer-s-disease.986.en.html?tid=1138&phlex_override_command=element
17	A05-19D	Non-drug therapies in Alzheimeŕs disease
	2009	https://www.iqwig.de/a05-19d-non-drug-therapies-in-alzheimer-s-disease.986.en.html?tid=1136&phlex_override_command=element
18	A05-20A	Selective serotonin and norepinephrine re-uptake inhibitors (SNRI) in the treatment of depression
	2009	https://www.iqwig.de/a05-20a-selective-serotonin-and-norepinephrine-re.986.en.html?tid=1134&phlex_override_command=element
19	A05-20C	Bupropion, mirtazapine, and reboxetine in the treatment of depression
	2009	https://www.iqwig.de/a05-20c-bupropion-mirtazapine-and-reboxetine-in.986.en.html?tid=1132&phlex_override_command=element
20	A05-21A	Weight reduction in essential hypertension
	2006	https://www.iqwig.de/a05-21a-weight-reduction-in-essential-hypertension.986.en.html?tid=1131&phlex_override_command=element
21	A05-22	Inhaled insulin (Exubera) in diabetes mellitus - rapid report
	2006	https://www.iqwig.de/a05-22-inhaled-insulin-exubera-in-diabetes.986.en.html?tid=1118&phlex_override_command=element
22	A05-23	Exenatide in diabetes mellitus type 2 - Rapid report
	2007	https://www.iqwig.de/a05-23-exenatide-in-diabetes-mellitus-type-2.986.en.html?tid=1117&phlex_override_command=element
23	A07-01	Fixed combinations of corticosteroids and long-acting beta-2-receptor agonists for inhaled use in patients with asthma - supplementary commission
	2008	https://www.iqwig.de/a07-01-fixed-combinations-of-corticosteroids-and.986.en.html?tid=1114&phlex_override_command=element
24	A08-01	Rapid-acting insulin analogues in children and adolescents with diabetes mellitus type 1 - follow-up commission
	2009	https://www.iqwig.de/a08-01-rapid-acting-insulin-analogues-in-children.986.en.html?tid=1112&phlex_override_command=element
25	A09-03	Update search on Report A05-19A (cholinesterase inhibitors in the treatment of Alzheimer's Disease) - Rapid report
	2009	https://www.iqwig.de/a09-03-update-search-on-report-a05-19a.986.en.html?tid=1113&phlex_override_command=element
26	A09-04	Drug treatment of hypertension - update search (rapid report)
	2010	https://www.iqwig.de/a09-04-drug-treatment-of-hypertension-update.986.en.html?tid=1246&phlex_override_command=element
27	D06-01A	Positron emission tomography (PET) in malignant lymphoma
	2009	https://www.iqwig.de/d06-01a-positron-emission-tomography-pet-in.986.en.html?tid=1135&phlex_override_command=element
28	D07-01	Osteodensitometry in primary and secondary osteoporosis
	2010	https://www.iqwig.de/d07-01-osteodensitometry-in-primary-and-secondary.986.en.html?tid=1122&phlex_override_command=element
29	N04-01	Non-drug local procedures in the treatment of benign prostatic hyperplasia
	2008	https://www.iqwig.de/n04-01-non-drug-local-procedures-in-the-treatment.986.en.html?tid=1200&phlex_override_command=element
30	N04-02	Interstitial brachytherapy in localized prostate cancer
	2007	https://www.iqwig.de/n04-02-interstitial-brachytherapy-in-localized.986.en.html?tid=1196&phlex_override_command=element
31	N04-03	Negative pressure wound therapy
	2006	https://www.iqwig.de/n04-03-negative-pressure-wound-therapy.986.en.html?tid=1198&phlex_override_command=element
32	N04-04	Balneo-phototherapy
	2007	https://www.iqwig.de/n04-04-balneo-phototherapy.986.en.html?tid=1199&phlex_override_command=element
33	N05-01	Implant-supported supraconstructions for the treatment of shortened dental arches
	2009	https://www.iqwig.de/n05-01-implant-supported-supraconstructions-for.986.en.html?tid=1193&phlex_override_command=element
34	N05-02	Relevance of the condition of the opposite dentition when fitting a fixed or removable denture
	2009	https://www.iqwig.de/n05-02-relevance-of-the-condition-of-the-opposite.986.en.html?tid=1178&phlex_override_command=element
35	N05-03A	Stem cell transplantation for adults with acute lymphoblastic leukaemia (ALL) or acute myeloid leukaemia (AML)
	2007	https://www.iqwig.de/n05-03a-stem-cell-transplantation-for-adults-with.986.en.html?tid=1177&phlex_override_command=element
36	N05-03B	Stem cell transplantation for severe aplastic anaemia
	2007	https://www.iqwig.de/n05-03b-stem-cell-transplantation-for-severe.986.en.html?tid=1181&phlex_override_command=element
37	N05-03D	Autologous stem cell transplantation for soft tissue sarcoma
	2009	https://www.iqwig.de/n05-03d-autologous-stem-cell-transplantation-for.986.en.html?tid=1182&phlex_override_command=element
38	N05-03E	Autologous stem cell transplantation for breast cancer
	2009	https://www.iqwig.de/n05-03e-autologous-stem-cell-transplantation-for.986.en.html?tid=1183&phlex_override_command=element
39	N05-03F	Unrelated donor allogeneic stem cell transplantation for Hodgkin's lymphoma
	2010	https://www.iqwig.de/n05-03f-unrelated-donor-allogeneic-stem-cell.986.en.html?tid=1184&phlex_override_command=element
40	N06-01A	Hyperbaric oxygen therapy for burns
	2007	https://www.iqwig.de/n06-01a-hyperbaric-oxygen-therapy-for-burns.986.en.html?tid=1171&phlex_override_command=element
41	N06-01D	Hyperbaric oxygen therapy for idiopathic osteonecrosis of the femoral head in adults
	2007	https://www.iqwig.de/n06-01d-hyperbaric-oxygen-therapy-for-idiopathic.986.en.html?tid=1174&phlex_override_command=element
42	N06-02	Negative pressure wound therapy - rapid report
	2007	https://www.iqwig.de/n06-02-negative-pressure-wound-therapy-rapid.986.en.html?tid=1157&phlex_override_command=element
43	N09-01	Non-drug local procedures for treatment of benign prostatic syndrome - Update - rapid report
	2010	https://www.iqwig.de/n09-01-non-drug-local-procedures-for-treatment-of.986.en.html?tid=1123&phlex_override_command=element
44	Q05-01A	Volume of operations and the quality of outcome for elective surgery of an abdominal aortic aneurysm
	2006	https://www.iqwig.de/q05-01a-volume-of-operations-and-the-quality-of.986.en.html?tid=1235&phlex_override_command=element
45	Q05-01B	Volume of operations and the quality of outcome for PTCA
	2006	https://www.iqwig.de/q05-01b-volume-of-operations-and-the-quality-of.986.en.html?tid=1236&phlex_override_command=element
46	S05-01	Neonatal screening for early detection of hearing impairment
	2007	https://www.iqwig.de/s05-01-neonatal-screening-for-early-detection-of.986.en.html?tid=1179&phlex_override_command=element
47	S05-02	Screening for visual impairment in children
	2008	https://www.iqwig.de/s05-02-screening-for-visual-impairment-in-children.986.en.html?tid=1180&phlex_override_command=element
48	S05-03	Ultrasound screening in pregnancy - test quality with regard to the detection rates of foetal abnormalities
	2008	https://www.iqwig.de/s05-03-ultrasound-screening-in-pregnancy-test.986.en.html?tid=1176&phlex_override_command=element
49	S06-01	Screening for defined speech and language development disorders in children
	2009	https://www.iqwig.de/s06-01-screening-for-defined-speech-and-language.986.en.html?tid=1140&phlex_override_command=element
50	S07-01	Screening for gestational diabetes
	2009	https://www.iqwig.de/s07-01-screening-for-gestational-diabetes.986.en.html?tid=1128&phlex_override_command=element
51	S07-01	Search update for report S07-01 - Screening for gestational diabetes
		https://www.iqwig.de/search-update-for-report-s07-01-screening-for.986.en.html?tid=1281&phlex_override_command=element
52	V06-02B	Interventions in young children with obstructive airway diseases
	2009	https://www.iqwig.de/v06-02b-interventions-in-young-children-with.986.en.html?tid=1232&phlex_override_command=element
53	V06-02C	Scientific evaluation of different investigational methods used in diagnosing “bronchial asthma” in children aged 2 to 5 years
	2009	https://www.iqwig.de/v06-02c-scientific-evaluation-of-different.986.en.html?tid=1233&phlex_override_command=element
54	V07-01	Relationship between volume of services and outcome in the care of preterm infants and neonates with very low birth weight (VLBW)
	2008	https://www.iqwig.de/v07-01-relationship-between-volume-of-services.986.en.html?tid=1224&phlex_override_command=element

**Table 2:** Appendix 2. Reports excluded.

	Project No	Title
	Year	Link
1	A05-21B	Reduction of salt intake in essential hypertension - Rapid report
	2009	https://www.iqwig.de/a05-21b-reduction-of-salt-intake-in-essential.986.en.html?tid=1129&phlex_override_command=element
2	B05-01A	Calculation of threshold values for minimum volumes for total knee joint endoprosthesis
	2006	https://www.iqwig.de/b05-01a-calculation-of-threshold-values-for.986.en.html?tid=1218&phlex_override_command=element
3	B05-01B	Calculation threshold values for minimum volumes in coronary surgery
	2006	https://www.iqwig.de/b05-01b-calculation-threshold-values-for-minimum.986.en.html?tid=1217&phlex_override_command=element
4	G05-01A	Development of a prognosis model to identify effects of threshold values on health care
	2006	https://www.iqwig.de/g05-01a-development-of-a-prognosis-model-to.986.en.html?tid=1216&phlex_override_command=element
5		Pilot study: Analytic Hierarchy Process in the indication “major depression”
	2010-01-05	https://www.iqwig.de/pilot-study-analytic-hierarchy-process-in-the.986.en.html?tid=1409&phlex_override_command=element
6		Guideline synopsis on depression
	2009-07-21	https://www.iqwig.de/guideline-synopsis-on-depression.986.en.html?tid=1241&phlex_override_command=element
7		Knowledge, perceptions and attitudes of German GPs concerning IQWiG, the Federal Joint Committee (G-BA) and EBM
	2009-07-15	https://www.iqwig.de/knowledge-perceptions-and-attitudes-of-german-gps.986.en.html?tid=1289&phlex_override_command=element
8	P04-01	A methodological proposal for developing IQWiG patient information
	2005	https://www.iqwig.de/p04-01-a-methodological-proposal-for-developing.986.en.html?tid=1214&phlex_override_command=element
9	P05-05A	Evidence-based patient information on chronic obstructive airway diseases – COPD
	2007	https://www.iqwig.de/p05-05a-evidenzbasierte-patienteninformationen.986.html?tid=1212&phlex_override_command=element
10	P05-05B	Evidence-based patient information on chronic obstructive airway diseases – Asthma
	2008	https://www.iqwig.de/p05-05b-evidenzbasierte-patienteninformationen.986.html?tid=1213&phlex_override_command=element
11	P05-06	Fact sheet for pregnant women on HIV tests
	2007	https://www.iqwig.de/p05-06-fact-sheet-for-pregnant-women-on-hiv-tests.986.en.html?tid=1207&phlex_override_command=element
12	P06-01	Expertise on endometriosis
	2008	https://www.iqwig.de/p06-01-expertise-on-endometriosis.986.en.html?tid=1206&phlex_override_command=element
13	V06-01	Quality of haematological and oncological care in children
	2009	https://www.iqwig.de/v06-01-quality-of-haematological-and-oncological.986.en.html?tid=1234&phlex_override_command=element
14	V-06-02A	Standard for diagnosis of bronchial asthma in young children
	2008	https://www.iqwig.de/v06-02a-standard-for-diagnosis-of-bronchial.986.en.html?tid=1231&phlex_override_command=element
15	V06-03	Systematic guideline search and appraisal for the DMP “CHD”
	2008	https://www.iqwig.de/v06-03-systematic-guideline-search-and-appraisal.986.en.html?tid=1230&phlex_override_command=element
16	V06-04	Systematic guideline search and appraisal for the DMP “Asthma/COPD” Project
	2009	https://www.iqwig.de/v06-04-systematic-guideline-search-and-appraisal.986.en.html?tid=1229&phlex_override_command=element
17	V06-05	Systematic guideline search for the DMP “Breast cancer”
	2008	https://www.iqwig.de/v06-05-systematic-guideline-search-for-the-dmp.986.en.html?tid=1227&phlex_override_command=element
18	V06-06	Systematic guideline search and appraisal for the DMP “Obesity”
	2009	https://www.iqwig.de/v06-06-systematic-guideline-search-and-appraisal.986.en.html?tid=1228&phlex_override_command=element
19	V09-01A	Exploration of the topic “Decompression for carpal tunnel syndrome” - Rapid report
	2009	https://www.iqwig.de/v09-01a-exploration-of-the-topic-decompression.986.en.html?tid=1220&phlex_override_command=element
20	V09-01B	Exploration of the topic “Conization of the cervix uteri” - Rapid report
	2009	https://www.iqwig.de/v09-01b-exploration-of-the-topic-conization-of.986.en.html?tid=1221&phlex_override_command=element
21	V09-01C	Exploration of the topic “Cataract surgery” -Rapid report
	2009	https://www.iqwig.de/v09-01c-exploration-of-the-topic-cataract-surgery.986.en.html?tid=1222&phlex_override_command=element
22	V09-01	Exploration of the topic “Surgery for varices” - Rapid report
	2009	https://www.iqwig.de/v09-01d-exploration-of-the-topic-surgery-for.986.en.html?tid=1223&phlex_override_command=element
23	2008-01-22	Working paper. Unrelated donor stem cell transplantation acquired severe aplastic anaemia Project
		https://www.iqwig.de/unrelated-donor-stem-cell-transplantation.986.en.html?tid=1215&phlex_override_command=element
24		Determination of relevant changes in oral health status
	2006-10-31	https://www.iqwig.de/determination-of-relevant-changes-in-oral-health.986.en.html?tid=1285&phlex_override_command=element
25		ebm@school - Development of a curriculum to impart basic health literacy competency to school pupils
	2006-10-31	https://www.iqwig.de/ebm-school-development-of-a-curriculum-to-impart.986.en.html?tid=1287&phlex_override_command=element
26		Association between nursing capacity and quality of outcome in inpatient care
	2006-08-07	https://www.iqwig.de/association-between-nursing-capacity-and-quality.986.en.html?tid=1240&phlex_override_command=element
37		Working paper: Evaluation of the benefits and harms of statins (with particular consideration of atorvastatin)
	2006-02-14	https://www.iqwig.de/evaluation-of-the-benefits-and-harms-of-statins.986.en.html?tid=1204&phlex_override_command=element

In total, 1087 clinical studies were included in these 54 reports (20.1 studies per report on average). Six reports included more than 50 studies. Of all studies, 843 were assigned to 45 countries while 193 studies (18%) were multinational. 51 studies (5%) were classified as unknown, which was mainly due to the fact that the IQWiG reports lacked information on the country of origin [19], [20].

### Number of clinical studies by countries

**Figure 1:**
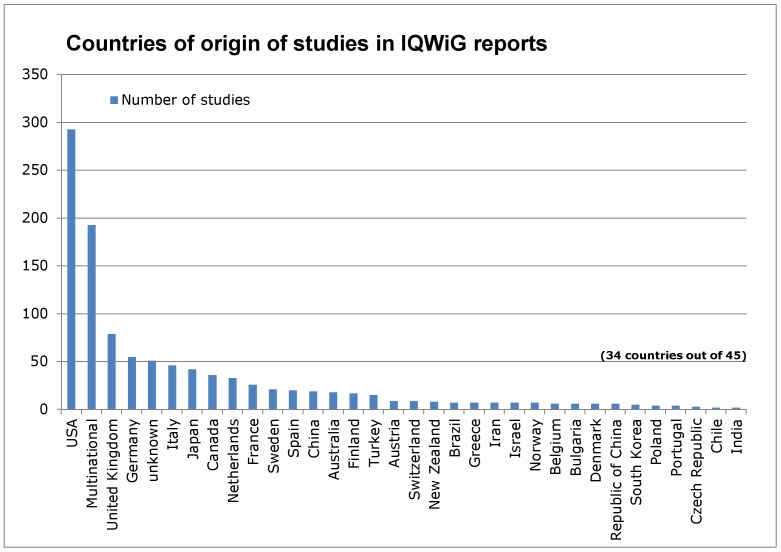
Countries of origin of studies in IQWiG reports.

Figure 1 displays the absolute number of studies included in IQWiG HTA reports per country of origin, which shows that the United States (USA) led the ranking (293 studies, 27%). Following in descending order were: 193 multinational clinical studies (18%), the United Kingdom (UK, 79 studies, 7%), and Germany (55 studies, 5%).

### Clinical studies by countries adjusted by population, GDP, and health expenditure

**Figure 2:**
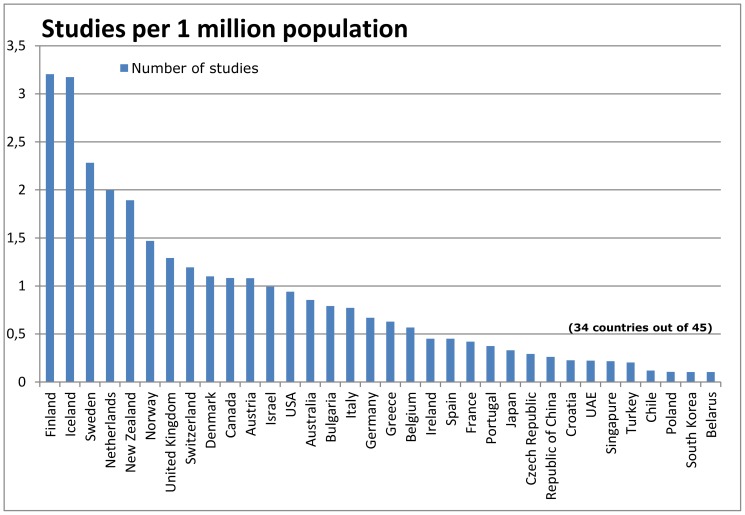
Number of studies in relation to population.

After adjusting the absolute number of studies by population size, Finland (3.21 studies per 1 million population) ranked first, followed by other Nordic countries (figure 2). The UK (1.29) dropped to position 7, the USA (0.94) and Germany (0.67) to positions 13 and 17 (figure 2).

**Figure 3:**
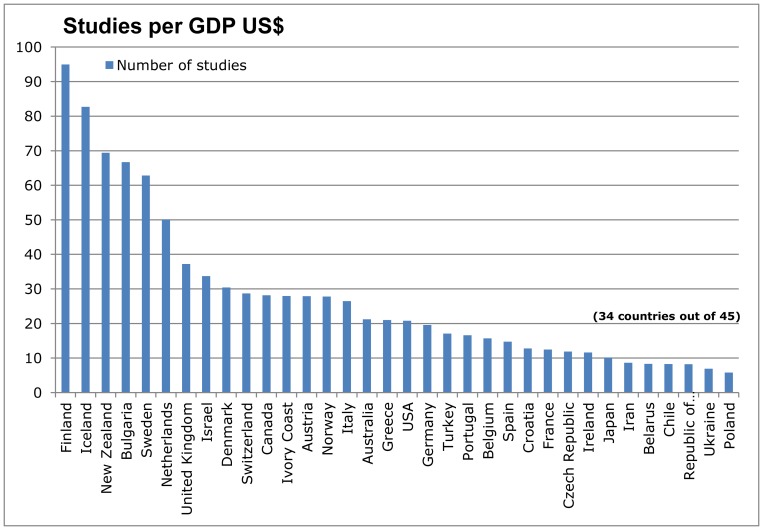
Number of studies in relation to Gross Domestic Product.

When using GDP to measure and adjust for a country's wealth, Finland (95.0 studies per billion US$ GDP) and Iceland (82.7) led the ranking. Other Nordic countries followed on ranks 5, 9 and 14 (62.8-27.80). The UK (37.2) was still at 7th position while the USA (20.8) and Germany (19.6) dropped to ranks 18 and 19. Bulgaria rose to 4th position (66.7) (figure 3).

**Figure 4:**
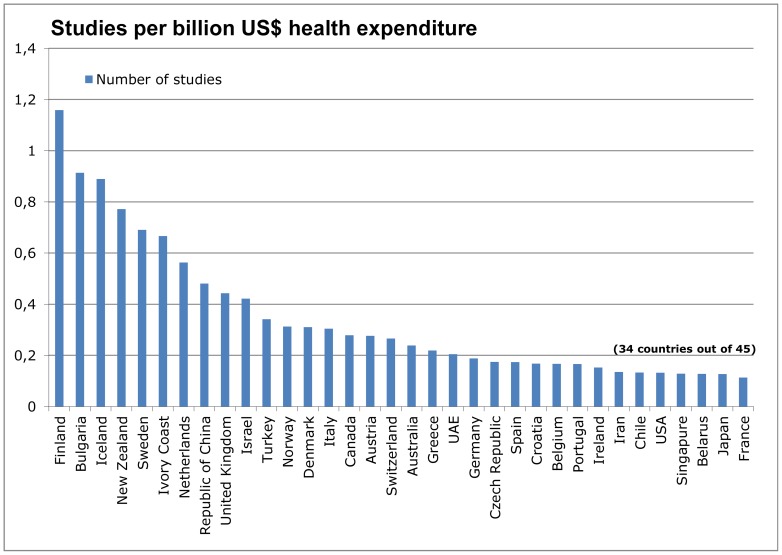
Number of studies in relation to health expenditure.

Adjusted for health expenditure, Finland (1.2 studies per 1 billion US$ health expenditure) again ranked first, followed by Bulgaria (0.91). Other Nordic countries followed on ranks 3, 5, 12 and 13 (0.88–0.31). The Ivory Coast moved up to sixth position (0.67), the UK (0.44) dropped to position 9, Germany (0.19) to 21 and the USA (0.13) to position 30 (figure 4).

### Influence of research topic

**Figure 5:**
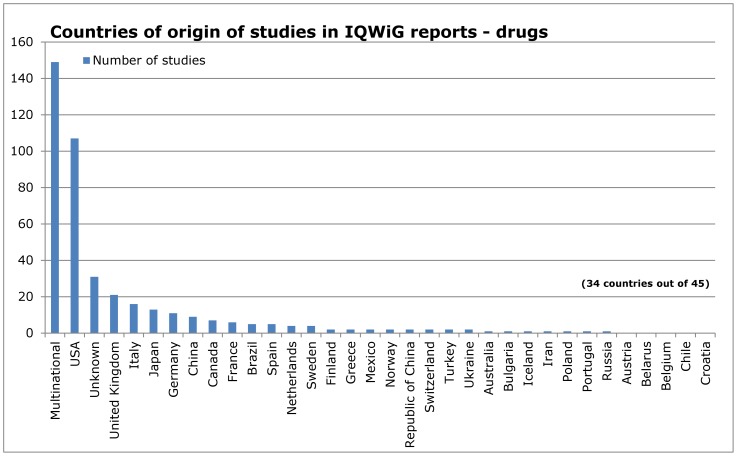
Countries of origin of studies in IQWiG reports – drugs.

**Figure 6:**
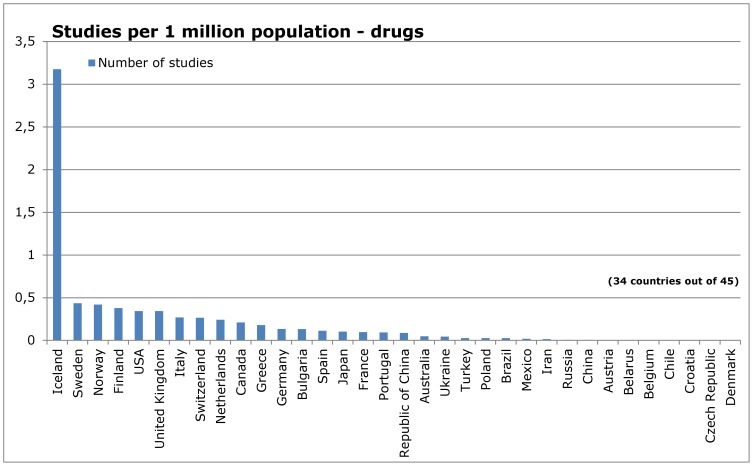
Number of studies in relation to population – drugs.

**Figure 7:**
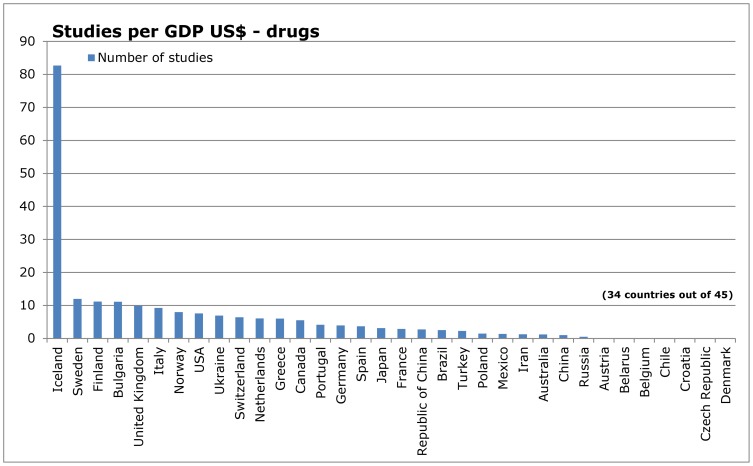
Number of studies in relation to Gross Domestic Product – drugs.

**Figure 8:**
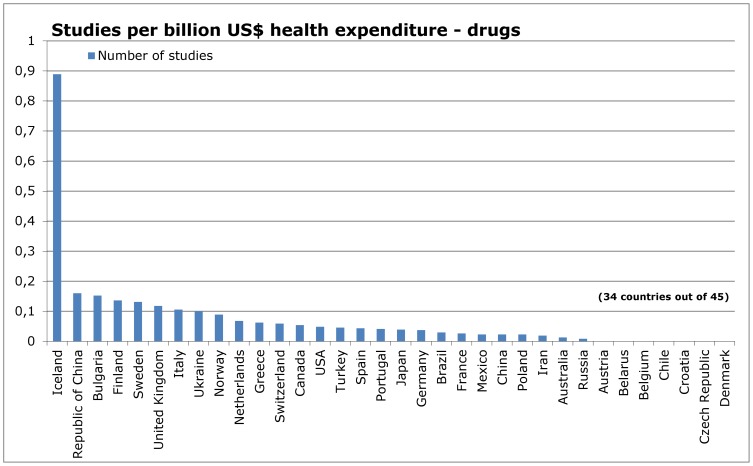
Number of studies in relation to health expenditure – drugs.

**Figure 9:**
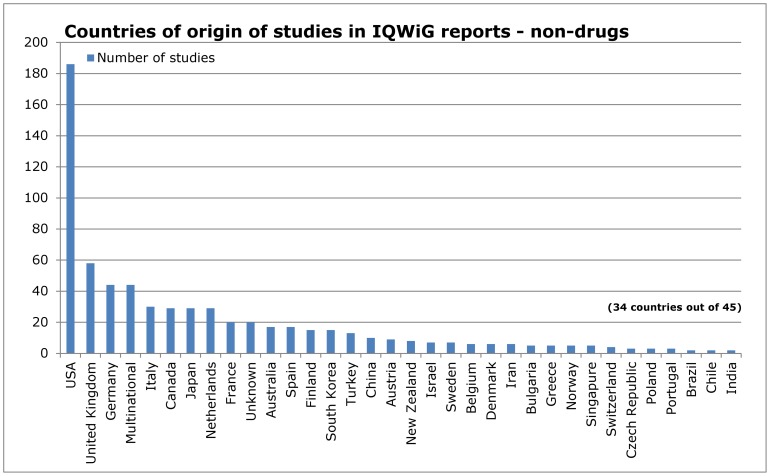
Countries of origin of studies in IQWiG reports – non- drug interventions.

**Figure 10:**
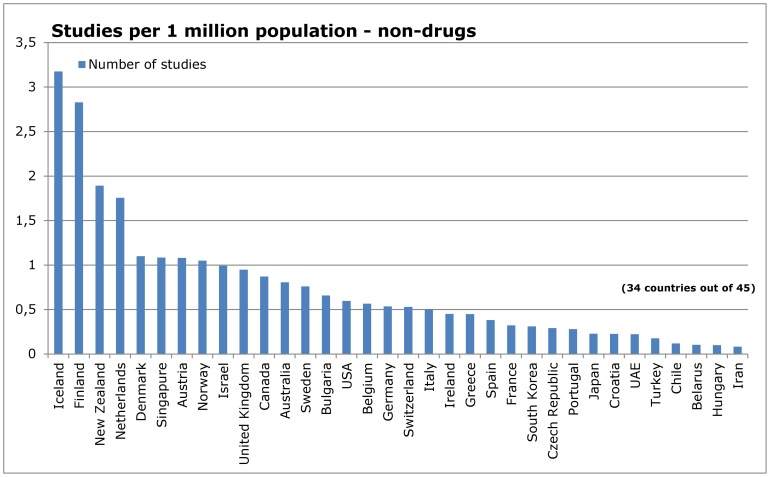
Number of studies in relation to population – non-drug interventions.

**Figure 11:**
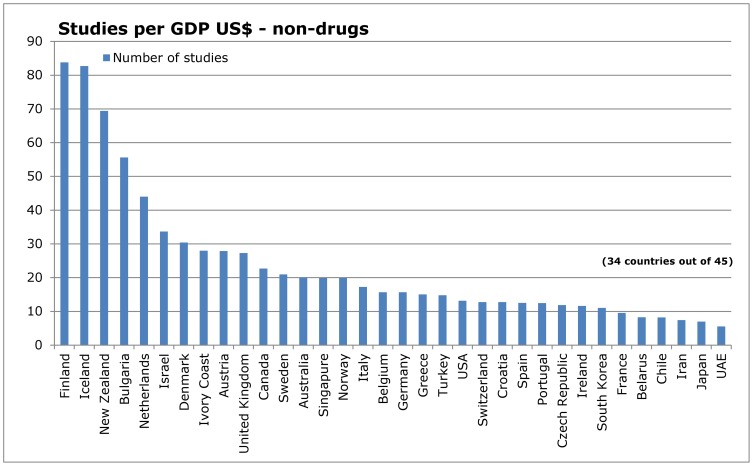
Number of studies in relation to Gross Domestic Product – non-drug interventions.

**Figure 12:**
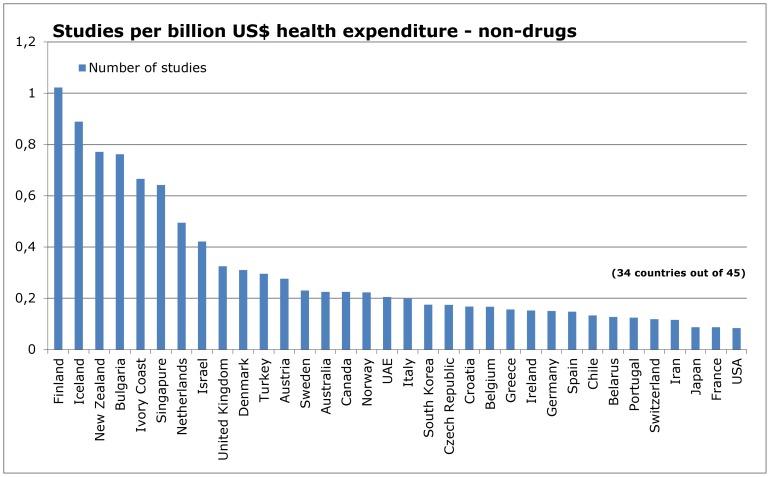
Number of studies in relation to health expenditure – non-drug interventions.

When dividing the reports by type of intervention (figure 5 – 12), most of the multinational studies and studies with unknown country of origin were on drugs (multinational 149, unknown 31, out of 410 studies in total); fewer were on non-drug interventions (multinational 44, unknown 20, out of 677 studies in total) (figure 5 and 9).

When adjusted for health expenditure, most countries remained stable in their ranking (figure 8 and 12). Only 6 of 28 countries with sufficient data showed differences of more than 20 places in the ranking of research on drugs and non-drug interventions. China, ROC, Ukraine and USA were more productive in drug research, whereas New Zealand and South Korea were more productive in non-drug research (table 3).

**Table 3:** Appendix 3. Comparison Drug- versus Non-Drug Studies per billion US$ health expenditure.

Country	Drug Studies	Non-Drug Studies	Difference in ranks
	per billion US$ health expenditure ranks	per billion US$ health expenditure ranks	
Iceland	1	2	Minor
**Republic of China**	2	-	Large
Bulgaria	3	4	Minor
Finland	4	1	Minor
Sweden	5	13	Minor
United Kingdom	6	9	Minor
Italy	7	18	Moderate
**Ukraine**	8	-	Large
Norway	9	16	Minor
Netherlands	10	7	Minor
Greece	11	23	Moderate
Switzerland	12	(30)	Moderate
Canada	13	15	Minor
**USA**	14	(34)	Large
Turkey	15	11	Minor
Spain	16	(26)	Moderate
Portugal	17	(29)	Moderate
Japan	18	(32)	Moderate
Germany	19	25	Minor
Brazil	20	-	Moderate or large*
France	21	33	Moderate
Mexico	22	-	Moderate or large*
**China**	23	-	Moderate or large
Poland	24	-	Moderate or large*
Iran	25	(31)	Minor
**New Zealand**	-	3	Large
Ivory Coast	-	5	Large*
Singapore	-	6	Large*
Israel	-	8	Large*
Denmark	(34)	10	Large*
Austria	-	12	Large*
Australia	(26)	14	Moderate
UAE	-	17	Moderate or large*
**South Korea**	-	19	Moderate or large
Czech Republic	(33)	20	Moderate
Croatia	(32)	21	Moderate
Belgium	(30)	22	Minor
Ireland	-	24	Moderate or large* (Sparse data)
38 Countries			Minor: 12
			Moderate: 10
			Large: 6
			Sparse data: 10

* Sparse data =  10 or less studies in either of the two groups.

Difference in ranks minor: 0 – 10; moderate10 – 20; large ≥ 20.

## Discussion

HTA reports are important for translating research into policy-making. They aim to inform policy-making comprehensively and with a minimized risk of bias. In order to do so, the global pool of clinical studies, often described as the “body of evidence”, has to be exploited. The objective of our study is to investigate the country of origin of clinical studies included in HTAs in a specific country, using Germany as an example. The results reflect the national contributions with a particular focus on research relevant to supporting health policy, as HTA is defined as a policy support tool, and within this framework reports are produced to provide answers to relevant questions.

### Summary of findings

The findings of our analysis confirm the leading role of the USA and UK as major contributors to the global pool of clinical studies providing relevant information for health-policy decisions. These are followed by a large proportion of multinational studies. Germany contributes only 5% of the research input included in IQWiG reports. When adjusted for population size or economic variables, Nordic countries dominate the ranking, while the relevance of the USA and Germany decreases noticeably. After adjustments, the position of the UK is more stable than that of the USA and Germany.

One limitation in the present analysis is that neither studies of unknown origin nor multinational studies were analyzed in depth, since data on the distribution of countries in the study reports were not reported in detail in the IQWIG reports. We stratified studies from drug and non-drug reports and found that most of the multinational studies were within the pool of drug studies. This might be explained by the fact that to be granted widespread approval for the same drug, a pharmaceutical company must submit approval studies to different regulatory authorities in different countries applying different legislation. It therefore makes sense to conduct large multinational studies in a variety of countries. Different and less stringent regulations apply for the approval of non-drug interventions.

Germany performs poorly compared with other countries of similar economic power. This finding is especially surprising as one would expect German HTA reports to include a higher proportion of German studies, as clinical research in a given country is more likely to address the same research questions of relevance as investigated in national HTA reports (e.g. for demographic or epidemiologic reasons). The underrepresentation of German studies might be caused by limited clinical research activity or by a lower output of studies relevant to health policy decision-making.

Overall, our data show vast differences between contributing countries. These differences are particularly striking when adjusted for country population, GDP or health care expenditure, i.e. showing national contributions per capita or per money unit. Rich countries such as Germany show a poor contribution to the global knowledge pool, which is in sharp contrast to the dependence of these countries on global knowledge for decision-making.

It should be noted that regardless of the size of a contribution, all countries are dependent on knowledge generated globally. In countries such as the USA, which contributes a large number of studies to the knowledge pool (in our analysis: 27%), users of information might be tempted to base their decision-making process on their ‘own’ trials. However, succumbing to this temptation is likely to cause serious problems. First, ignoring large parts of the available evidence is a waste of resources and would introduce bias, as decision-making in health care should be based on all of the available evidence. Second, stratification for medical specialties would change the country league tables considerably, in some fields even dramatically [6]. The obvious conclusion is that all countries should consider themselves as contributors to and beneficiaries from the global body of evidence.

### Research results in context

Comparison of the present analysis to previous ones largely confirmed earlier findings of studies comparing national activities in patient-oriented research. There are many similarities, regardless of whether an analysis was based on studies cited by Cochrane reviews [6], [21], pharmacological trials [11], or high-ranking publications in primary care [12], surgery [14], anesthesia [7], [8], nuclear medicine [10], or dentistry [9]; Nordic and Anglo-American countries usually take the lead.

Gluud and Nikolova [21] described various factors that have to be taken into account to explain a country's scientific output. Population size, economic wealth and research expenditure are obvious and relatively simple factors to include, while historical and cultural aspects are more difficult to cover. Factors with a strong impact are the research expenditure of pharmaceutical companies, as well as collaboration between researchers and industry. National government budgets play a key role in the funding of clinical research, as well as in the regulation of RCTs on drugs, especially the time taken to obtain regulatory approval [11].

There are numerous other factors possibly explaining the lack of clinical studies; for example, the promotion policies of clinical research, the funding situation, and the specific requirements concerning the availability of specially trained and experienced medical and research staff might be very different from those in other countries [12], [22]–[24].

Numerous hypotheses for future testing can be derived from our analysis. It would be interesting to see whether our findings are supported by similar research in other countries. In addition, the cultural component should be investigated, as well as other questions related to decision science, for example, when to use national or multinational studies or foreign studies from similar or very different countries.

### Strengths and limitations

Our analysis differs from existing approaches. We did not merely conduct a bibliometric analysis, but investigated which countries contributed the largest proportion of studies included in HTAs in Germany. Studies had thus undergone a rigorous quality assessment according to IQWiG methods [5]. At the same time our analysis considered the relevance of the research output. All 1087 studies assessed by IQWiG to inform health-policy decisions were included, reflecting the performance of different countries in producing research relevant to decision-making.

One limitation in the present analysis was that neither studies with unknown origin nor multinational studies were analyzed in depth, since data on the distribution of countries in the study reports were not reported in detail in the IQWIG reports.

Some results need to be interpreted with caution. As an example, when weighted by national health expenditures, the Ivory Coast reached sixth place. This is rather misleading, as only one study [25] was included in an HTA report on test accuracy in ultrasound screening in pregnancy [26]. However, due to very low national health expenditure, the Ivory Coast achieved this relatively high ranking.

There might potentially be other factors associated with the output of clinical trials than population size, GDP, or health expenditure, such as promotion policies within departments, the number of universities, their programs, and funding in this field [12], [24], which were not analyzed in our study.

## Conclusion

According to our findings, there is a discrepancy between the use of globally generated evidence and the contribution to the knowledge pool by individual countries. In absolute numbers, by far the most studies relevant to evidence-informed decision-making in Germany were conducted in the USA, followed by multinational research and the UK.

From the perspective of contributing countries, absolute numbers are misleading as they imply contributions, which do not exist on a per capita level but are merely due to a “large country effect”. Our study confirms that some small countries have a remarkable input in relation to their population size, health expenditure, or GDP. In contrast, some larger rich countries profit from these imbalances. Germany belongs to this category, with only 5% of the studies in German HTA reports actually conducted in Germany. Even for countries with larger contributions, it would be unwise to ignore the globally available evidence, and even harmful in certain fields because of much richer information outside their own countries.

The often-noted and criticized lack of studies for many relevant clinical questions is a consequence of many countries not taking the responsibility to contribute to global knowledge on the same scale as they are benefitting from it. Limited resources are a crucial issue in all research fields. In the medical field the existing gaps in knowledge are not a pure research problem but have a serious impact on health care decisions on an individual and public health level. A better-balanced contribution of all countries to the generation of global knowledge and its translation into policy and practice are urgently required to eradicate these deficits [27].
